# Scapula Kinematics of Youth Baseball Players

**DOI:** 10.1515/hukin-2015-0107

**Published:** 2015-12-30

**Authors:** Gretchen Oliver, Wendi Weimar

**Affiliations:** 1 Auburn University. School of Kinesiology. Auburn, Alabama. USA

**Keywords:** youth athlete, injury prevention, kinetic chain, upper extremity

## Abstract

Literature has revealed the importance of quantifying resting scapular posture in overhead athletes as well as quantifying scapular kinematics during dynamic movement. Prior to this project much of the attention in throwing research had been focused on the position of the humerus without description of the positioning of the scapula. Therefore, it was the purpose of this study to present scapular kinematics during pitching in youth baseball players. Twenty-five youth baseball players (age 11.3 + 1.0 years; body height 152.4 + 9.0 cm; body mass 47.5 + 11.3 kg), with no history of injury, participated in the study. Scapular kinematics at the events of maximum humeral external rotation (MER) and maximum humeral internal rotation (MIR) during the pitching motion were assessed three-dimensionally while pitching fastballs for strikes. Results revealed that at the event of MER, the scapula was in a position of retraction, upward rotation and a posterior tilt. While at the event of MIR, the scapula was protracted, upward rotated and tilted anteriorly.

## Introduction

The scapula plays a pivotal role in normal upper extremity movement. In addition, it is the major link in the proximal (lower extremity) to distal (upper extremity) transfer of velocity, energy and forces (Kibler, 1998). Any type of abnormality in scapular stability as well as movement may result in decreased performance, upper extremity pathomechanics, and possibly compensatory movements in the lower extremity. Though this information is known, there are no data regarding this performance during dynamic movements such as youth baseball pitching.

Scapular dyskinesis has been defined as any observable alteration in scapular position or motion (Kibler, 2010). These types of alterations have often been observed in baseball pitchers (Laudner et al., 2007; Meyer et al., 2008; Myers 2005; Myers et al., 2013). The mechanics of baseball pitching requires efficient utilization and coordination of the lower extremity, hips, pelvis, torso, and upper extremity. It is the scapula that must not only provide a stable base of support but also perform efficient movements, however, literature in throwing has yet to establish this function in dynamic movement. The scapula is an integral link between the lower and upper extremity as it funnels the energy created by the lower to the upper extremity through its timing and coordination of movement. The coordinated movement of the scapula is dependent upon the demand of humeral positioning, funneling energy, and as a stable platform for muscle attachment. However, the application of scapular coordinated movement and dynamic shoulder function is lacking.

During throwing, the scapula must be able to fully retract to allow for the proper cocking position, and then fully protract throughout the acceleration phase (Kibler, 1998). In addition to the coordination of retraction and protraction, the scapula also must efficiently upwardly rotate and elevate to allow for adequate glenohumeral function when the humerus is abducted to 90º (Kibler, 1998). The scapula not only allows for coordinated glenohumeral movement, it is also a muscle attachment site for the primary muscles controlling glenohumeral movement. Improper positioning of the scapula alters the line of pull of all the muscles acting upon the glenohumeral joint. If the scapula is not functioning succinctly with the humerus, glenohumeral joint integrity and range of motion are compromised (Kibler, 2010; Kibler et al., 2013; Laudner et al., 2007; Meyer et al., 2013).

Alterations in scapular positioning have been implicated in shoulder injury (Myers, 2005). In addition, scapular dyskinesis has often been associated as a contributor to shoulder injury in throwing athletes. With the high association of scapular function and injury, research has examined scapula positioning and movement during humeral elevation (Amasay and Karduna, 2009; Laudner et al., 2007; Mihata et al., 2012; Myers et al., 2013). However, there has yet to be a study examining scapular orientation during the dynamic movement of throwing. While this investigation is important to all throwers, it is paramount to young throwers. With the cumulative incidence, in youth pitchers, of a 5% risk of sustaining an upper extremity injury within a 10-year period (Fleisig et al., 2011), scapular orientation and positioning could assist in quantifying potential injury pathomechanics as well as provide the basis for performance enhancing mechanics. It was therefore our purpose to present the first documentation of scapular kinematics during pitching in youth baseball players; particularly, scapular kinematics at the events of maximum humeral external rotation (MER) and maximum humeral internal rotation (MIR) during the pitching motion. We hypothesized that scapular kinematics quantified during youth baseball pitching would exhibit scapular retraction and upward rotation at MER and scapular protraction and upward rotation at MIR.

## Material and Methods

### Participants

Twenty-five youth baseball players (age 11.3 + 1.0 years; body height 152.4 + 9.0 cm; body mass 47.5 + 11.3 kg) participated in the study. The selection criteria included coach recommendation, years of pitching experience (at least two years), and no injury within the past six months. Coach recommendation was sought to ensure that the participants were experienced pitching competitively for at least two years. Moreover, none of the participants reported that they had ever suffered an injury to their throwing arm. Nor did any participants suffer from any pain or stiffness in their upper or lower extremity following extensive throwing sessions. The Auburn University’s Institutional Review Board approved all testing protocols. Prior to data collection all testing procedures were explained to each participant and their parent(s)/legal guardian(s) and informed consent and participant assent were obtained. All participants were tested during their fall throwing season and had not thrown prior to their arrival for testing.

### Procedures

The MotionMonitor™ (Innovative Sports Training, Chicago IL) synchronized with an electromagnetic tracking system (Flock of Birds Ascension Technologies Inc., Burlington, VT) was used to collect data. The electromagnetic tracking system had been validated for tracking humeral movements, producing trial-by trial interclass correlation coefficients for axial humerus rotation in both loaded and non-loaded conditions in excess of 0.96 (Ludewig, 2000b). With electromagnetic tracking systems, field distortion was shown to be the cause of error in excess of 5º at a distance of 2m from an extended range transmitter (Day, 2000), but increases in instrumental sensitivity reduced this error to near 10º prior to system calibration and 2º following system calibration (Meskers, 1999; Perie, 2002). Thus, prior to data collection, the current system was calibrated using previously established techniques (Day, 2000; Meskers, 1999; Perie, 2002). Following calibration, pilot data collected prior to testing participants indicated that the magnitude of error in determining the position and orientation of the electromagnetic sensors within the calibrated world axes system was less than 0.01 m and 3º, respectively.

Participants had a series of 11 electromagnetic sensors affixed to the skin using PowerFlex cohesive tape (Andover Healthcare, Inc., Salisbury, MA) to ensure the sensors remained secure throughout testing. Sensors were attached to the following locations: (1) the posterior/medial aspect of the torso at C7, (2) posterior/medial aspect of the pelvis at S1, (3–4) bilateral distal/posterior aspect of the upper arm, (5) the flat, broad portion of the acromion of the scapula, (6–7) bilateral distal/posterior aspect of the forearm, (8–9) bilateral distal/posterior aspect of the lower leg, and (10–11) bilateral distal/posterior aspect of the upper leg (Myers, 2005; Myers et al., 2013; Oliver and Keeley, 2010; Oliver and Plummer, 2011; Oliver, 2013). A twelfth sensor was attached to a stylus that was used for digitization of the bony landmarks described by the International Shoulder Group of the International Society of Biomechanics (Wu et al., 2005). Two points described the longitudinal axis of each segment and the third point defined the plane of the segment. A second axis was defined perpendicular to the plane and the third axis was defined as perpendicular to the first and second axes. Participants stood in the anatomical position during digitization to guarantee accurate bony landmark identification. The digitization of the bony landmarks allowed transformation of the sensor data from the global coordinate system to the anatomically based local coordinate system (Table 1). The neutral stance was the y-axis in the vertical direction, horizontal and to the right of y was the x-axis, and posterior was the z-axis (Oliver and Plummer, 2011). Data describing the position and orientation of electromagnetic sensors were collected at 100 Hz. Raw data were independently filtered along each global axis using a 4^th^ order Butterworth filter with a cutoff frequency of 13.4 Hz (Oliver and Keeley, 2010a; 2010b). Reliability of the scapulohumeral kinematic protocol used in the current study had been previously presented (intraclass correlation coefficient, 0.63–0.96) (Thigpen et al., 2005).

Euler angle decompositions were used to determine scapular (Y, X′ Z″) and humeral (Y, X′, Y″) orientation with respect to the thorax. The first scapular rotation was defined around the y-axis (internal (+)/external rotation (−)). Rotation about the y-axis is also referred to as scapular protraction (internal rotation) and retraction (external rotation). The second scapular rotation was about the x-axis (upward (+)/downward rotation (−)), and the third scapular rotation was defined as rotation about the z-axis (anterior (−)/posterior tilting (+)). Anterior tilting was defined when the inferior angle of the scapula moved away from the thorax. Humeral angles were also defined relative to the thorax. Humeral orientation was determined as rotation about the y-axis of the humerus (plane of elevation), rotation about the z-axis (elevation), and rotation about the y-axis (axial rotation) (Table 2). Plane of elevation defined as 0º was abduction and 90º was forward flexion. Each of the aforementioned rotations was based on the recommendations of the International Shoulder Group (Wu et al., 2005).

Once the sensors were attached, the participant was given an unlimited time to perform their own specified pre-competition warm-up routine. Average warm-up time was 10 min. Once the participants deemed themselves warm, they were instructed on the protocol. They were required to throw maximal effort four-seam fastballs for strikes over a regulation distance (46 feet; 14.02 m) to a catcher. A JUGS radar gun (OpticsPlanet, Inc., Northbrook, IL) positioned in the direction of the throw determined ball speed. The fastest of the four-seam fastballs for strikes was selected for analysis (Barrentine et al., 1998; Oliver and Keeley, 2010a; 2010b; Werner et al., 2005).

### Statistical Analysis

The scapular position and orientation were analyzed at the events of MER and MIR of the pitching motion. Data were analyzed for descriptive statistics of means and standard error of the mean using IBM SPSS Statistics 20 (IBM corp., Armonk, NY). The dependent variables measured were scapular kinematics (internal/external rotation, upward/downward rotation, and anterior/posterior tilt) at the pitching events of MER and MIR.

## Results

Scapular kinematic data (means and standard error of the mean) are presented for positions of rest, MER and MIR in Table 3. At the point of MER, the scapula was in a position of retraction/external rotation, upward rotation and a posterior tilt. While at the event of MIR, the scapula was protracted/internally rotated, upward rotated and anteriorly tilted.

## Discussion

The purpose of this study was to present the first documentation of scapular kinematics during pitching in youth baseball players; particularly, scapular kinematics at the events of MER and MIR during the pitching motion. The results indicate that at the event of MER, the scapula was in a position of retraction, upward rotation and a posterior tilt. While at the event of MIR, the scapula was protracted, upward rotated and tilted anteriorly. The results of the current study are similar to previously published literature of the scapular position at 90º of humeral elevation (Amasay et al., 2009; Laudner et al., 2013; Myers et al., 2005; Seitz et al., 2012) (Table 4).

A major role of the scapula is retraction, to allow for the shoulder to be in a position of approximately 90º of abduction and maximum external rotation in the cocking phase of the baseball pitch (Kibler, 1998) as in our study the event of MER. Scapular retraction allows for greater tension on the anterior musculature, thus, subsequently changing from eccentric at MER to concentric as the arm accelerates into internal rotation for ball release and MIR. A reduction in scapular retraction at MER may result in impingement susceptibility as well as an increased load placed on the rotator cuff musculature, in addition to inefficient compensatory patterns of the lower extremity (Kibler et al., 2013).

In addition to scapular retraction at MER, the scapula also was in a position of upward rotation and a posterior tilt. A decrease in upward rotation is thought to result in a decrease in subacromial space as the humerus is abducted (Laudner et al., 2007; Ludewig 2000a). Decreased scapular upward rotation may result in decreased efficiency of the kinetic chain between the upper and lower extremities, decreased muscular function, and subsequent risk of injury (Kibler, 1998; Kibler et al., 2013; Pain and Voight, 1993). Upward rotation of the scapula allows for adequate subacromial space when the humerus is in the position of 90º of abduction and thus needed for efficient pitching mechanics (Laudner et al., 2007). Additionally, a posterior tilt functionally has been suggested to allow for additional subacromial space as the humerus is abducted (McClure et al., 2001), thus allowing for a more optimal position at the pitching event of MER.

Efficient acceleration of the shoulder into internal rotation requires scapular protraction (Kibler, 1998). As the acceleration proceeds, the scapula must protract laterally and anteriorly around the thoracic wall in an effort to maintain glenohumeral congruity as well as assist in force dissipation (Kibler, 1998) through the event of MIR and on to the follow through phase (Thomas et al., 2010). In the current study, the scapula was in a position of protraction/internal rotation, upward rotation, and an anterior tilt at the event of MIR. Though the scapula was in a position of upward rotation, in relation to the upward rotation at MER, it was greatly decreased. The combination of decreased scapular upward rotation and an anterior tilt results in decreased subacromial space during shoulder elevation (Kibler et al., 2013; Laudner et al., 2006; Ludewig 2000a; Provencher et al., 2014).

The current study presents the position of the scapula at two events of MER and MIR during pitching in youth. The current data are in agreement with previous studies examining scapular kinematics at the position of humeral elevation at 90º. Though only few have examined baseball pitchers (Myers et al., 2007; Laudner et al., 2013), we were able to have comparable values to others. Prior to this project much of the attention in the throwing literature had been focused on the position of the humerus without description of the positioning of the scapula. Furthermore, it is the first time that scapular kinematics during youth baseball pitching has been presented. This project is the first in a long line of research aiming to investigate the influence of scapular positioning on glenohumeral dynamics, ball speed and pelvic girdle motion. It is hoped that these data will aid in the more complete understanding of glenohumeral mechanics and potentially pathomechancis during overhead motions.

The authors of this study recognize a couple of methodological limitations that must be addressed. First, motion analysis using sensors attached to the skin are susceptible to skin artifact. To account for this we only examined positions below 120º of arm elevation, as kinematic data of the scapula are reliable in humeral elevation angles below 120º (Karduna et al., 2001). Additionally, the protocol used had previously presented an intraclass correlation coefficient of 0.63–0.96 (Thigpen et al., 2005). During pitching the position of MER is the point of maximum humeral external rotation and 90º of humeral elevation (Provencher et al., 2014; Kibler et al, 1998), thus not exceeding the humeral elevation of 120º. Additionally, youth pitching data have revealed that humeral elevation is within the range of 95.8º + 12.7º (Keeley et al., 2012). Second, humeral kinematics were not considered in this analysis, however, kinematic data of the humerus were used to event mark the positions of MER and MIR. The authors choose to focus solely on scapular kinematics as the scapula is the base upon which humeral motion occurs and as such is the attachment site for many of the muscles that help control the humerus. In order to better understand humeral movement, one must first understand the position of the scapula so that lines of muscle force can be identified and applied to this understanding.

Understanding the position of the scapula during overhead throwing can assist coaches, strength and conditioning specialists and sports medicine personnel in appropriate pre-throwing exercises to target the musculature about the scapula. With repetitive throwing, scapular kinematics may become susceptible to injury, thus focusing on scapular stability as a precursor to throwing could possibly assist in maintaining scapular kinematics during throwing.

## Figures and Tables

**Table 1:** Description of bony landmarks palpated and digitized

Bony Landmark	Bony Process Palpated & Digitized
**Thorax**	
Seventh Cervical Vertebra (C7)	Most dorsal aspect of the spinous process
Eighth Thoracic Vertebra (T8)	Most dorsal aspect of the spinous process
Suprasternal Notch	Most cranial aspect of sternum
**Scapula**	
Angulus acromialis	Most lateral-dorsal point of the scapula
Trigonum spine	Midpoint of triangular surface on medial boarder of scapula in line with scapular spine
Angulus inferior	Most caudal point of scapula
**Humerus**	
Medial Epicondyle	Medial/distal aspect of condyle
Lateral Epicondyle	Lateral/distal aspect of condyle
Glenohumeral Joint Center of Rotation	Rotation method*

* The center of glenohumeral rotation was not digitized.

The rotation method estimated the joint center using least of squares algorithm for the point moving the least during a series of short rotational movements.

**Table 2:** Angle orientation decomposition sequences

Segment	Axis of Rotation	Angle
**Scapula**		
Rotation 1	Y	Internal (+)/External Rotation (−)*
Rotation 2	X′	Upward (+)/Downward Rotation (−)
Rotation 3	Z″	Anterior (−)/Posterior Tilt (+)
**Shoulder**		
Rotation 1	Y	Plane of Elevation (0=Abduction; 90=Flexion)
Rotation 2	X′	Elevation
Rotation 3	Y″	Internal Rotation (+)/External Rotation (−)

* Internal rotation is also referred as protraction, external rotation also referred to as retraction.

Prime (′) and double prime (″) notations represent previously rotated axes due to the rotation of the local coordinate system resulting in all axes within that system being rotated.

**Table 3:** Scapular Kinematic Data Expressed in Degrees

Kinematic Variable	Mean	Standard Error of Mean
**Anatomical Neutral**		
Internal/External Rotation	−26.6	14.3
Upward/Downward Rotation	2.2	7.0
Anterior/Posterior Tilt	−14.5	7.8
**Maximum External Rotation (MER)**		
Internal/External Rotation	21.6	15.2
Upward/Downward Rotation	36.6	11.6
Anterior/Posterior Tilt	13.5	14.7
**Maximum Internal Rotation (MIR)**		
Internal/External Rotation	−19.6	12.3
Upward/Downward Rotation	23.4	13.3
Anterior/Posterior Tilt	−5.8	13.2

Internal Rotation [−]; External Rotation [+]; Upward Rotation [+]; Downward Rotation [−]; Anterior Tilt [−]; Posterior Tilt [+]

**Table 4:** Scapular Kinematic Data Reported in the Literature

	Kinematic Variable	Mean	SD/SEM
**Humeral Elevation 90º**			
Myers et al., 2005	External Rotation	45.6	10.28 (SD)
	Upward Rotation	26.9	7.08 (SD)
	Posterior Tilt	−8.57	8.7 (SD)
Laudner et al., 2013	Upward Rotation	22.2	6.7 (SD)
Amasay et al., 2009	External Rotation	27.4	11.2 (SD)
	Upward Rotation	26.9	4.5 (SD)
	Posterior Tilt	−1.6	8.9 (SD)
	External Rotation	36.0	1.7 (SEM)
Seitz et al., 2012	Posterior Tilt	−8.2	1.7 (SEM)

All data are expressed in degrees
